# Dislocation luminescence in GaN single crystals under nanoindentation

**DOI:** 10.1186/1556-276X-9-649

**Published:** 2014-12-01

**Authors:** Jun Huang, Ke Xu, Ying Min Fan, Jian Feng Wang, Ji Cai Zhang, Guo Qiang Ren

**Affiliations:** 1 Suzhou Institute of Nano-tech and Nano-bionics, CAS, Suzhou 215123, People’s Republic of China; 2 Suzhou Nanowin Science and Technology Co., Ltd., Suzhou 215123, People’s Republic of China

**Keywords:** GaN, Nanoindentation, Dislocation, Luminescence

## Abstract

This work presents an experimental study on the dislocation luminescence in GaN by nanoindentation, cathodoluminescence, and Raman. The dislocation luminescence peaking at 3.12 eV exhibits a series of special properties in the cathodoluminescence measurements, and it completely disappears after annealing at 500°C. Raman spectroscopy shows evidence for existence of vacancies in the indented region. A comprehensive investigation encompassing cathodoluminescence, Raman, and annealing experiments allow the assignment of dislocation luminescence to conduction-band-acceptor transition involving Ga vacancies. The nanoscale plasticity of GaN can be better understood by considering the dislocation luminescence mechanism.

## Background

GaN-related III-nitride materials have gained an unprecedented attention due to their wide-ranging applications such as short-wavelength optoelectronic devices [1], high-electron-mobility transistor [2], and semiconductor lasers [3]. However, due to the lack of large-sized bulk materials, the majority of GaN-related alloys or structures are grown heteroepitaxially on foreign substrates such as sapphire or SiC. Consequently, those alloys or structures usually contain a high density of dislocations which can have detrimental effects on the performance of devices. In spite of the considerable progress made in the last decade in GaN, an in-depth understanding of the properties of dislocation is needed due to their paramount importance in the growth of most conventional semiconductor materials and in the manufacture of semiconductor devices. However, the optical and electronic properties of as-grown dislocations may be greatly affected by the unintentionally introduced impurities and defects during the growth process. Thus, it is interesting to clarify intrinsic optical properties of dislocations both in basic research and technological applications.

Nanoindentation is an ideal technique for studying the fundamental behaviors and properties of dislocations in a crystal by introducing dislocations into a small volume that is initially defect-free. Consequently, nanoindentation experiments and simulations can be used to demonstrate mechanisms governing dislocation nucleation in a broad range of fields and applications [4,5]. Especially, there has also been a considerable effort to determine the properties of plastic deformation in GaN epilayers and GaN bulk crystals using indentation techniques [6-14]. Local strain fields of the indentation have been studied by a micro-Raman spectroscopy [11,13], and the formation of contact-induced dislocations has been investigated via cathodoluminescence (CL) spectroscopy [6-9,11] and transmission electron microscopy (TEM) [6-8,10,12]. However, most of these earlier studies mainly focused on the microstructure of the indentation-induced dislocations in GaN; the fundamental dislocation luminescence mechanism of GaN is not understood fully. This work presents a comprehensive study encompassing nanoindentation, CL, and Raman techniques aimed at revealing the origin of the dislocation luminescence in GaN.

### Experimental details

A 1.5-mm-thick freestanding GaN layer with an area size of about 20 mm × 20 mm was selected for the indentation tests. The thick GaN layer grown by hydride vapor phase epitaxy on the c-plane of sapphire substrate was self-separated during cooling down from the growth temperature. The dislocation density of the GaN freestanding layer was about 5 × 10^5^ cm^−2^ as estimated by the etch pit density. The background carrier concentration was about 1 × 10^16^ cm^−3^ from the analysis of the Hall data.

Nanoindentation tests were performed on the GaN (0001) surface using a nanoindentation system (Nano Indenter G200, Agilent Technologies, Inc., Santa Clara, CA, USA). A Berkovich indenter tip with a radius of curvature of 50 nm was employed for indentation experiments. The strain rate was set at 0.05 s^−1^ during nanoindentation tests. Scanning electron microscopy (Quanta 400 FEG, FEI, Hillsboro, OR, USA) - cathodoluminescence (MonoCL3+, Gatan, Inc., Pleasanton, CA, USA) system was used to characterize the indentation. The Raman spectra measured by a LabRAM HR 800 spectrometer (LabRAM HR 800 spectrometer, HORIBA Scientific, Edison, NJ, USA) were excited with the 633.28-nm He-Ne laser allowing for a lateral resolution of better than 1 μm.

## Results and discussion

**Figure 1:**
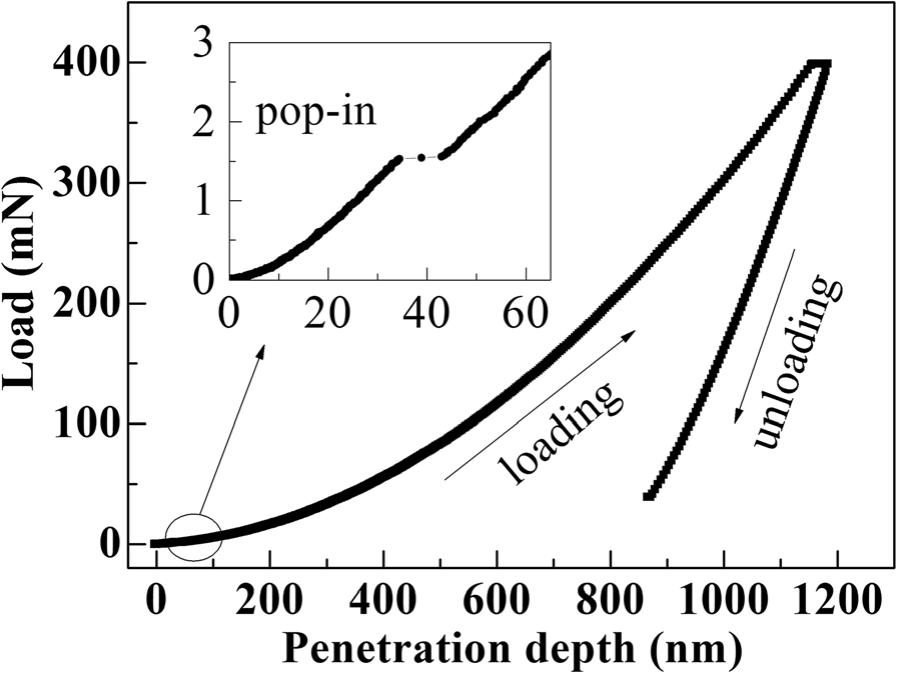
**Typical continuous load-penetration curves of GaN.** The maximum load is 398 mN. Inset: a magnification view of the pop-in event.

Figure 1 shows a typical load-penetration curve obtained from 1.5-mm-thick c-plane GaN loaded to a maximum of 398 mN. The unloading part of the load-penetration curve shows that the residual deformation depth of the indentation is about 865 nm. A sudden displacement discontinuity, the pop-in event, was observed in the loading part at 1.5 mN (see the inset in Figure 1). This phenomenon is attributed to dislocation nucleation and propagation during loading as have been observed in GaN [6-9].

**Figure 2:**
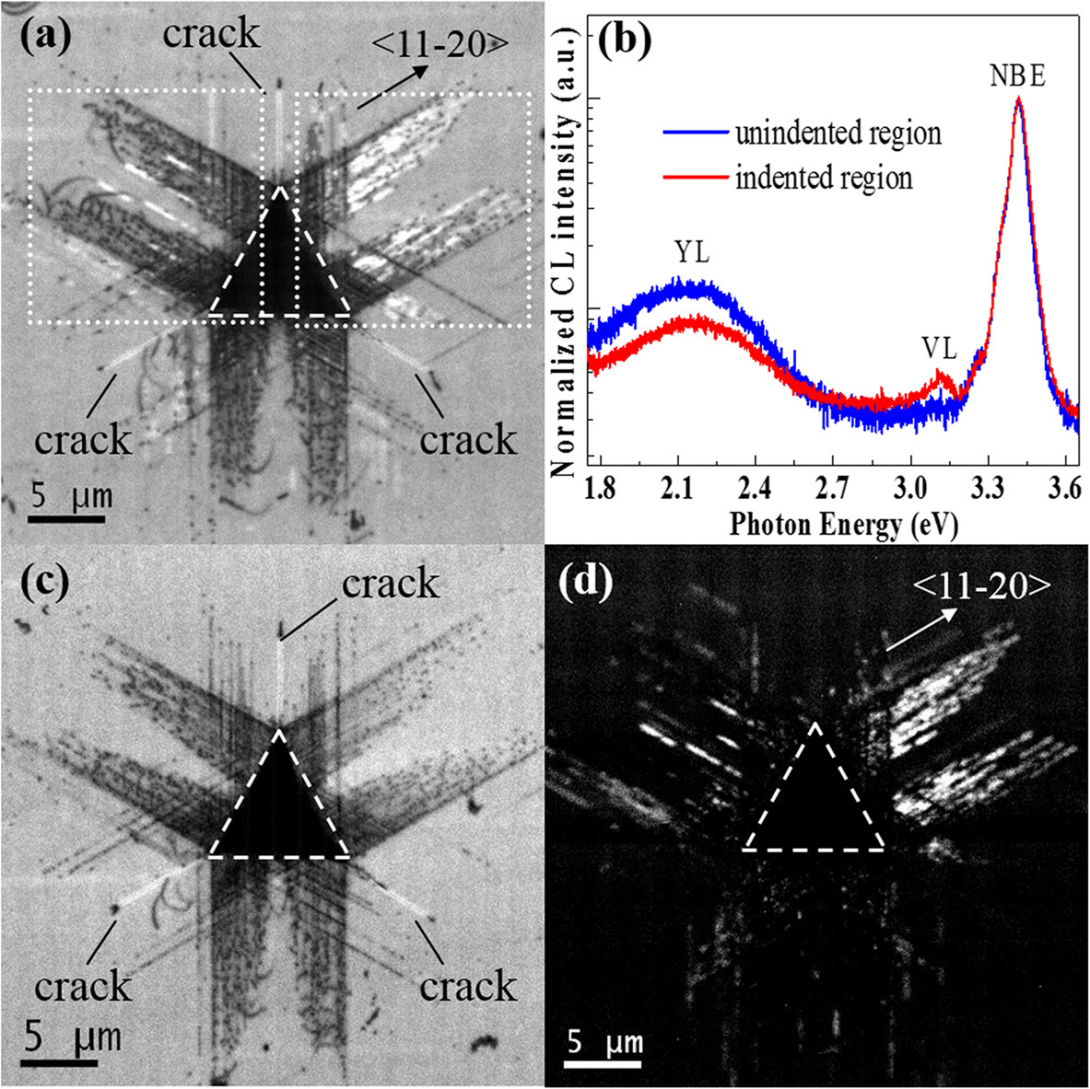
**CL images and spectra of a Berkovich indentation in c-plane GaN. (a)** Panchromatic CL image. **(b)** CL spectra of the indented region and the unindented region. **(c)** Monochromatic CL image collected at 3.40 eV photon energy. **(d)** Monochromatic CL image collected at 3.12 eV photon energy.

**Figure 3:**
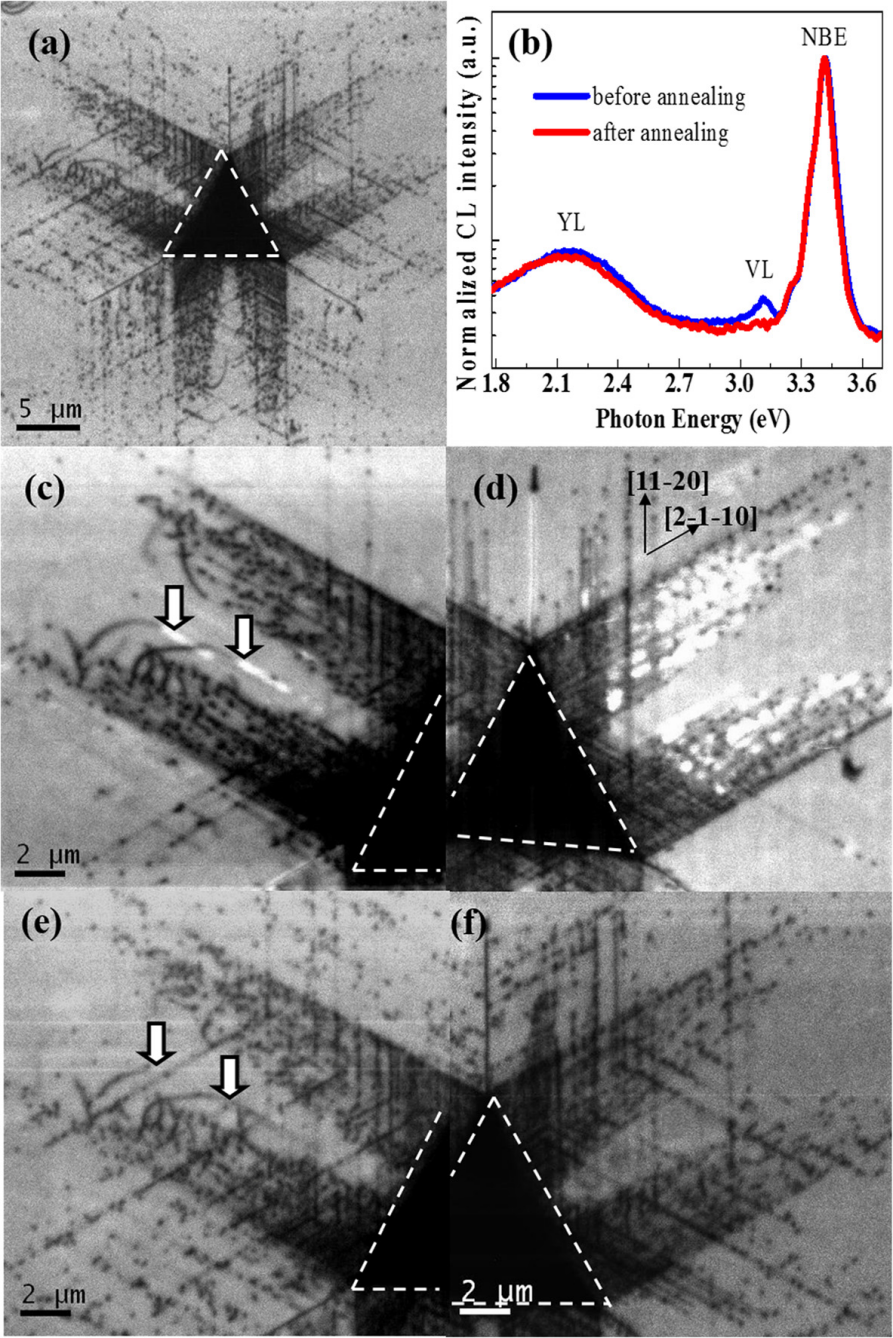
**Panchromatic CL images of the same indentation as shown in Figure **2**. (a)** Panchromatic CL image after annealing. **(b)** CL spectra of the indented region collected before and after annealing. **(c)** Magnified CL image of the top left corner region (denoted by the white dashed rectangle frame (left) in Figure 2a). **(d)** Magnified CL image of the top right corner region (denoted by the white dashed rectangle frame (right) in Figure 2a). **(e)** CL image of the same area of **(c)** collected after annealing. **(f)** CL image of the same area of **(d)** collected after annealing.

Figure 2a is a panchromatic CL image of an indentation in c-plane GaN. The indentation was made at a maximum load of 398 mN. The residual footprint of the indentation was marked out by a white dashed triangle line. The observed dark-line defects and dark-spot defects, which propagate radially out from the center of the nanoindentation along the <11-20 > crystal orientation at 60° intervals, are the different parts of the dislocation loops emerged on the free surface (for details, see Additional file 1 and reference [9]). In addition, cracks nucleated along the <11-20 > orientation were also found. Interestingly, bright luminescence around the dislocations is observed. In order to elucidate the origin of the bright luminescence, typical room temperature CL spectra normalized with respect to the band-edge peak are shown in Figure 2b. The spectrum collected from the indented region is characterized by three luminescence bands, namely near-band-edge emission (NBE) at about 3.40 eV, yellow luminescence (YL) around 2.20 eV, and the violet luminescence (VL) at about 3.12 eV. Notably, the YL band in the unindented region is stronger than in the indented region, indicating that some of the preexisting defects responsible for YL were driven out of the indented region by applying mechanical stress (the so call ‘mechanical annealing’, see reference [15]). Interestingly, the VL band can only be found in the indented region. In order to ascertain whether the VL band plays a dominant role in the bright luminescence around the dislocations, monochromatic CL images of the NBE band at 3.40 eV and the VL band at 3.12 eV of the same area of Figure 2a are shown in Figure 2c,d. In the CL image of Figure 2c obtained at 3.40 eV (NBE band), indentation-produced dislocations are clearly visible as dark regions where the intensity of the near-band-edge CL emission from GaN is dramatically suppressed due to nonradiative recombination. The CL image of Figure 2d obtained at 3.12 eV (VL band) clearly illustrates the distribution of optically active recombination centers which extend radially out from the center of the indentation along <11-20 > directions. Comparing the CL images of Figure 2c,d with the one of Figure 2a, the bright luminescence around the dislocations is the VL band centered at 3.12 eV. According to the previous reports, some types of dislocations were thought to be a radiative center in wurtzite GaN. Partial dislocation on the basal plane was found to be responsible for the strong emission at 3.14 eV in GaN [16]. Another work reported that the 60° dislocations of A type in the basal plane induce radiative transitions with energy at 2.90 eV in indented GaN [17]. In order to make this clear, we annealed the sample at 500°C for 60 min in an NH_3_ atmosphere.Interestingly, the high intensity emission around the indented dislocations in GaN disappears completely after annealing at 500°C as shown in Figure 3a, and the room temperature CL spectra of the indented region collected both before and after annealing confirm the quenching of VL (Figure 3b). Since the extend defects (stacking faults or dislocation) are not expected to be eliminated at such a low annealing temperature, they are not likely to be the origin of VL. This is further supported by panchromatic CL images of Figure 3c,f. In Figure 3c,d, the magnified CL images of the deformed regions denoted by white dashed rectangle frames in Figure 2a are shown. The VL seems to come from both the surrounding (denoted by arrows in Figure 3c) and the motion traces of dislocations (Figure 3d). In either case, the regions that emit VL extend exclusively out from the center of the indentations along the <11-20 > direction. After annealing at 500°C, some of the dislocations propagated away from the center of indentation, still many of them do not change their position as shown in Figure 3e,f (collected from the same regions of Figure 3c,d). Either way, the dislocations did not disappear with the quenching of VL after annealing. It confirms that the dislocation cannot be the source of VL.

In fact, the dislocations are widely thought to be a non-radiative center in wurtzite GaN; they are not likely to manifest themselves by luminescence, unless point defects are trapped at them [18]. Therefore, the most probable origin of VL is due to point defects.

**Figure 4:**
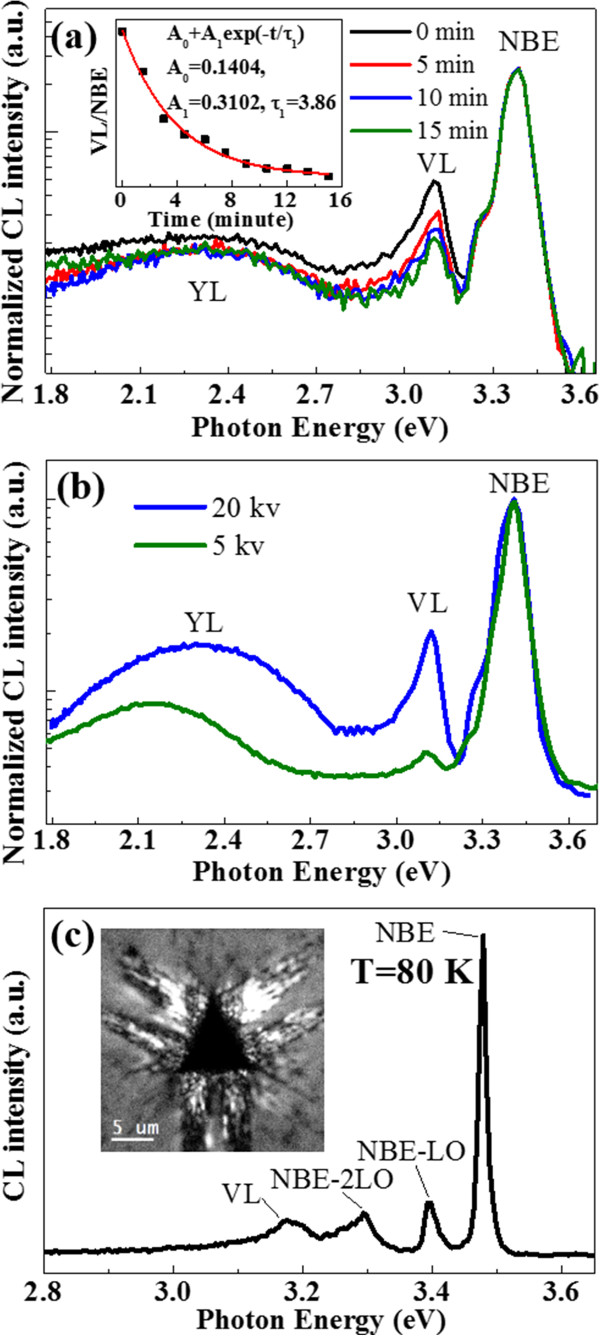
**CL spectra of an indentation in c-plane GaN. (a)** Evolution of intensity of 3.12 eV luminescence with exposure time exhibits exponential decay characteristic. The inset is the dependence of the intensity ratio of VL to NBE on exposure time, in which the dots are the experimental data and the solid line is the calculated curve with the formula in the inset. **(b)** CL spectra recorded at different values of acceleration voltage. **(c)** Low-temperature (80 K) CL spectrum.

Furthermore, VL band exhibits very special properties in the CL measurements, which may provide additional clues for the origin of the VL. Figure 4a shows time-dependent CL spectra of the indented region. Under a constant electron beam irradiation of 20 kV, the VL shows an attenuated behavior characteristic of metastable defect levels in GaN. The output intensity of the VL decreases with the exposure time while the output intensity of the NBE keeps constant. After 3.8 min of electron beam irradiation, the VL decays to less than one-half of the initial intensity. However, after moving away from the electron beam irradiation for several hours, the emission intensity of the VL of the same position is recovered to the initial intensity, which excludes the possibility that the attenuation of VL is due to irradiation damage. A similar metastable luminescence at 2.8 ~ 3.0 eV has been found in undoped GaN epilayers, which is thought to be related to a hole tarp center such as V_Ga_[19,20]. More information about the type of transition of the defects involved in the VL band can be obtained from CL spectra with variable excitation intensity. With the increasing excitation intensity (from 5 to 20 kV acceleration voltage), the peak position of the YL shifts evidently to higher energies as shown in Figure 4b. This is the distinguishing feature of the donor-acceptor-pair (DAP) recombination [18]. The nearly independence of the VL peak position on excitation intensity is a signature of the conduction-band-acceptor (e-A) transition [18]. However, the participation of the internal transitions in some defects cannot be excluded. Figure 4c shows a spectrum of the indented region collected at 80 K (inset is the monochromatic CL image of indentation obtained at a photon energy of 3.18 eV). Four luminescence bands, the NBE band at about 3.47 eV, the LO-phonon-assisted emission bands (NBE-LO, NBE-2LO) at about 3.39 and 3.29 eV, and the VL band at about 3.18 eV have been observed. It is well known that change of temperature induces shifts of CL bands. Due to the shift of VL (approximately 60 eV) that is similar to the shift of the band gap (approximately 70 eV), the VL can be attributed to e-A transitions [18]. Indeed, a much weaker temperature shift of a given CL band for internal transitions within the defect may be expected [21].

**Figure 5:**
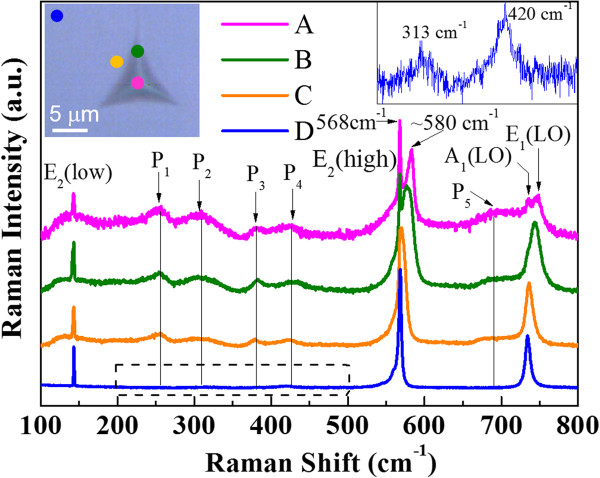
**Micro-Raman spectra for GaN outside, around, and different regions inside the indentation.** The spectra are plotted in logarithmic scale and displaced vertically for better viewing. Inset in the top left corner shows corresponding optical image of the indentation spot. Inset in the top right corner shows magnification of spectrum D in the range from 250 to 500 cm^−1^ (denoted by dashed rectangle frame).

Additionally, more information can be gained from an analysis of the Raman spectra of the indentation (see Figure 5). The inset in the top left corner of Figure 5 shows the spots measured outside, around, and inside the indentation recorded in the optical microscope attached to the spectrometer. Three main peaks centered at about 143, 568, 733 and 745 cm^−1^ are correlated with the first-order vibrational modes of E_2_(low), E_2_(high), A_1_(LO), and E_1_(LO) for GaN, respectively. Note that the E_1_(LO) phonon mode, which is forbidden in the backscattering geometry for the (0001) plane according to the selection rule in the wurtzite crystal of GaN [22], is found in the indented region (see spectra A and B in Figure 5). It indicates the occurrence of recrystallization in the indented region [23]. In addition, the E_2_(high) phonon peak is split into two sub-peaks for the indented region. The 568 cm^−1^ signal originates from the bulk of the sample, and the approximately 580 cm^−1^ peak originates from the highly compress stress region inside the indentation. Notably, besides the E_2_, A_1_ and E_1_ phonon peaks, five additional broad peaks were found inside and around the indented region: peak P_1_ at about 257 ± 1 cm^−1^, peak P_2_ at about 310 ± 3 cm^−1^, peak P_3_ at about 380 ± 1 cm^−1^, peak P_4_ at about 423 ± 3 cm^−1^, and peak P_5_ at about 670 ~ 690 cm^−1^. In the presence of defects, the residual stress, which coexists with hydrostatic strain induced by the defects, can cause an additional shift and broadening of the P_1_-P_5_ peak. According to previous reports [22,24,25], P_1_ (approximately 260 cm^−1^) was ascribed to local vibrational modes (LVMs) due to vacancies or dislocations, P_2_ (approximately 300 cm^−1^) was assigned to V_Ga_, P_4_ (approximately 420 cm^−1^) was assigned to LVMs due to N or Ga vacancies, and peak P_5_ (approximately 670 cm^−1^) was assigned to disorder-activated Raman scattering (DARS). The peak P_3_ (approximately 380 cm^−1^) has not been reported before. We tentatively assign P_3_ to LVMs due to vacancies or dislocations because it can be observed only in the indented region. It should be noted that some previous reports assigned the peak at approximately 300 cm^−1^ to DARS [26]. However, as shown in the inset in the top right corner of Figure 5 (magnification of spectrum D in the range from 250 to 500 cm^−1^), the P_2_ (as well as P_4_) can also be found outside the indentation, thus the Raman modes at 310 ± 3 cm^−1^ cannot be attributed to DARS. Indeed, because of the gap between the acoustic- and the optical-phonon branches from 300 to 530 cm^−1^[27], the Raman modes at 310 ± 3 cm^−1^ are hard to be explained by DARS. Note that both the P_2_ and P_4_ in the indented region are much stronger than in the unindented region; the formation of vacancy (including V_Ga_ and V_N_) is likely to occur.

From the above discussion, one can glean the obvious that the VL is related to a native point defect introduced by the indentation. Among all the native point defects in GaN, V_Ga_ appears to be the best candidate, since the transition energy from the conduction band to the 0/− transition level of V_Ga_ is estimated at about 3.15 eV [18], which is very close to the photon energy of VL. In addition, V_Ga_ was found to anneal out in long-range migration processes at 500 to 600 K [28,29], which is consistent with the vanishing of VL in indented GaN after annealing at 500°C. The assignment of the VL peak to V_Ga_ is also supported by the Raman spectra, since the Raman spectra have found evidence for the existence of Ga vacancies in the indented region. Therefore, the most plausible cause for the VL is the V_Ga_.

**Figure 6:**
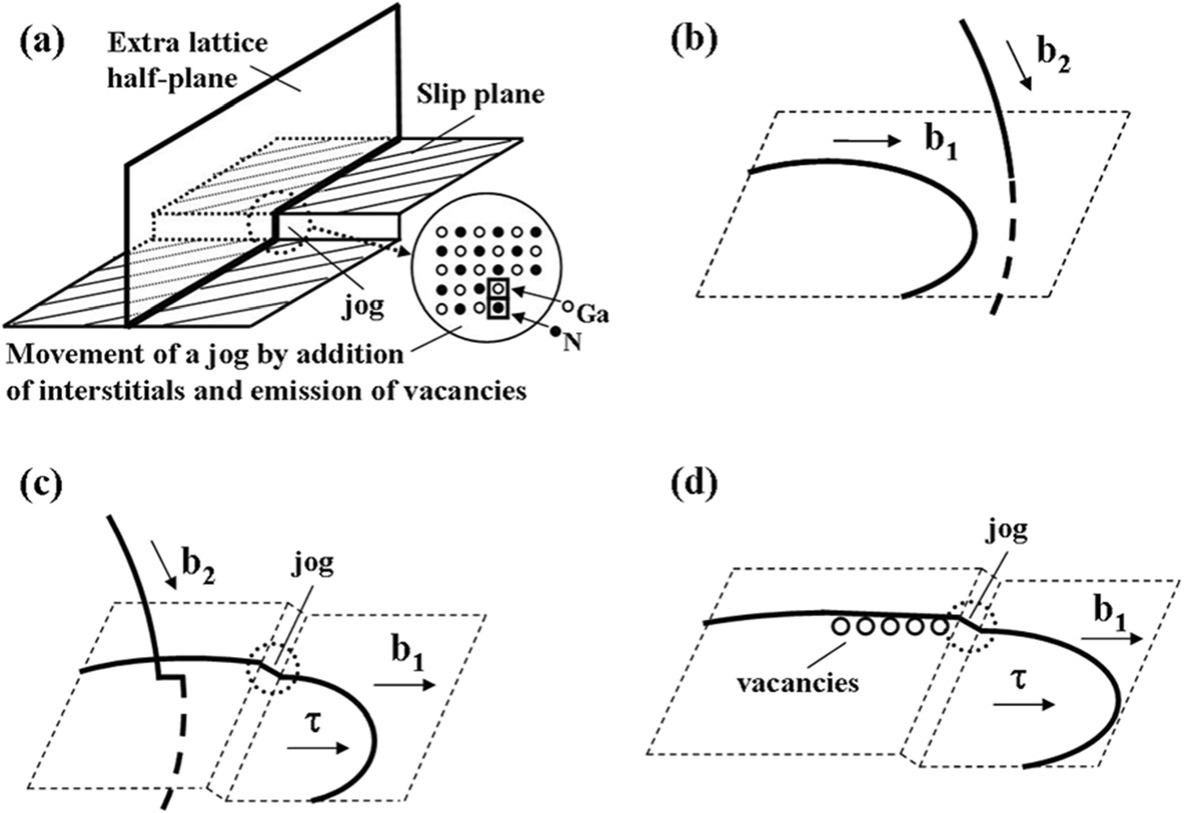
**The formation mechanism of vacancies by the motion of jogged dislocations. (a)** The jog is considered to be the step in a dislocation line with atomic dimensions. **(b,c)** The intersection of two dislocations with different Burgers vector produces one jog each per dislocation**,** and the jog which is out of slip plane would anchor the dislocation. **(d)** When the tension force of the dislocation line is large enough under applied stress, the jog taken by the dislocation can be forced to move forward through emission or absorption of vacancies.

In fact, the formation energies of vacancies and their complexes at different sites near the threading-edge dislocation are much lower than the formation energies of the corresponding defects in the bulk [30]. Energy levels of the vacancies trapped at dislocations generally shift as compared to the point defects in bulk; however, the shift is not large. The stress field of threading-edge dislocations is likely to trap Ga vacancies and their complexes. In addition, it is well know that the dislocation can climb by absorbing or emitting vacancies, and jogs in the dislocation line are the most favorable sites for these processes to occur. Based on the above analyses, a formation mechanism of vacancies by dislocation jogs movement is proposed and shown in Figure 6. A jog is a step in a dislocation line with atomic dimension that is not contained in the glide plane (Figure 6a). One jog in each per dislocation was produced after the intersection of two dislocations with different slip directions (Figure 6b). The jogs cannot move along the slip plane of the dislocation loop. Thus, the dislocation line which was driven forward by the shear stress would bend toward the slip direction under the pin of the jog (Figure 6c). Once the tension force of dislocation line exceeded a critical value, the jog was compelled to climb along the slip direction of the dislocation. Then a trail of vacancies aligning along <11-20 > direction (a slip direction of edge dislocation in GaN) appears in the wake of a jogged moving dislocation (Figure 6d). The formation mechanism of vacancies is consistent with the CL images presented above.

## Conclusions

In conclusion, the VL band peaking at about 3.12 eV from the region near the dislocations is characterized and identified. A comprehensive study encompassing CL measurements, annealing experiments, and Raman analysis allow the assignment of VL band to e-A transitions involving V_Ga_. A formation mechanism of vacancies by the motion of jogged dislocations is proposed to explain the dislocation luminescence in GaN single crystals under nanoindentation. The nanoscale plasticity of GaN can be better understood by considering that not only the dislocation mechanisms but also the nucleation of point defects are involved in the deformation.

## Competing interests

The authors declare that they have no competing interests.

## Authors’ contributions

KX designed and supervised the project. JH carried out all the experiments. YMF was involved in the nanoindentation experiments. JFW and GQR supervised the sample selection. JH and JCZ wrote the paper. All authors contributed to discussion of the results. All authors read and approved the final manuscript.

## Authors’ information

J. Huang is currently a Postdoctoral Associate in the Center of Characterization and Analysis, SINANO, CAS. K. Xu, J. F. Wang, J. C. Zhang, and G. Q. Ren are professors in the Center of Characterization and Analysis, SINANO, CAS. Y. M. Fan is a PhD student in the Center of Characterization and Analysis, SINANO, CAS.

## Supplementary Material

Additional file 1 **Sketch of dislocation loops emerging at the free surface.** The dark-line defects and dark-spot defects observed in the panchromatic CL image of the indentation are the different parts of dislocation loops emerged on the free surface. Click here for file
