# Supplementary figures and images for: Dislocation luminescence in GaN single crystals under nanoindentation
Source: Nanoscale Res Lett. 2014 Dec 1;9(1):649. doi: 10.1186/1556-276X-9-649 (PMC4275118; doi:10.1186/1556-276X-9-649)

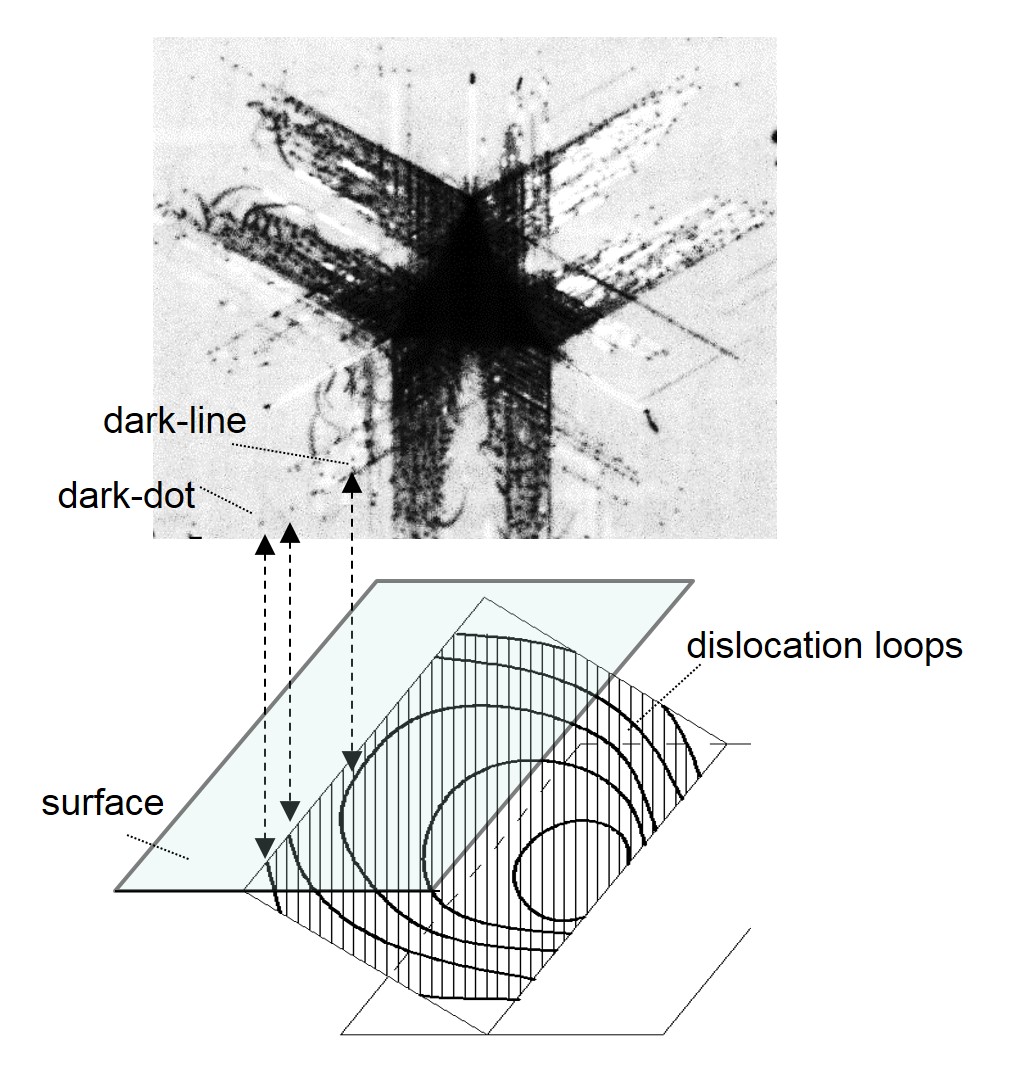

Supplement: Additional file 1 — Sketch of dislocation loops emerging at the free surface. The dark-line defects and dark-spot defects observed in the panchromatic CL image of the indentation are the different parts of dislocation loops emerged on the free surface. [file 1556-276X-9-649-S1.jpeg]
